# Prolapsus Uteri

**Published:** 1858-02

**Authors:** J. G. Westmoreland

**Affiliations:** Prof. of Materia Medica and Medical Jurisprudence, in the Atlanta Medical College, Atlanta, Georgia


### ARTICLE. III.

*Prolapsus Uteri.* By J. G. Westmoreland, M. D., Prof. of Materia Medica and Medical Jurisprudence, in the Atlanta Medical College, Atlanta, Georgia.

So diversified have been the views of physicians in regard to the true nature of this uterine displacement, that we are forced to conclude that very little is known of the pathological condition, or that the displacement is the result of disease of the uterus and its appendages, and may be found in different conditions of these organs. The latter, we may conclude, is a true solution of the diffi ulty. The uterus is maintained in its proper position by a healthy condition of the vagina, ligaments and the organ itself; and even then, in a subject of naturally lax fibre, sometimes slight displacements occur. As, then, the tendency of disease, such as chronic inflammation, ulceration, &c., in any part, is to destroy the tone, vigor and strength of the tissues implicated, it is reasonable to suppose that engorgement of the uterus, and the consequent irritation, inflammation and ulceration of this organ and the contiguous structures, will so destroy the natural tone and strength of those parts which support the organ in its normal position, that they no longer perform their functions properly, and prolapsus is the result. Again, any mechanical pressure from above, pressing upon the womb continually, may overcome the natural support, and bring about the precise state of things, so far as respects the position, as is found to exist from disease, as above stated. Indeed disease of these organs may be set up by such mechanical causes, concomitantly with the displacement; hence the great difficulty in many cases in determining the primary difficulty or source of the evil. One of the most fruitful mechanical causes of uterine disease and displacement, may perhaps be found in the habits of dress among females. I am frequently interrogated as to the cause of greater liability to uterine disturbance for the last few years than formerly, and am constrained to answer that the habit of suspending the most weighty of the clothing from the waist, subjects the abdominal and pelvic viscera to pressure in proportion to the amount of clothing thus worn, and from this cause the pressure is greater in the erect position, when the uterus from the force of gravitation is least calculated to resist it. The supposed increased frequency of female disease is accounted for in the change of the habits of that sex in other respects, such as exe:cise sleeping, &c. &c., but we find those who adopt the most approved hygienic course in these respects, not to any great extent exempt above those who subject themselves to a less enervating and tonic course of life. From whatever cause it may arise, there seems to be an increasing tendency to uterine disease, and in the absence of any change in the customs or habits of life among females likely to affect them in this respect, except that of dress, I can but conclude that to corsets and heavy skirts must be attributed the increased suffering in this particular.

From what has been said, it appears that the displacement which heads this article is only the result of other disease; but as this condition of the uterus, as regards position in the pelvis, has been set down among the catalogue of diseases, and as it has been thought of and treated under this name, it is proper to consider the causes of this displacement under it. Now, as the paramount consideration in the investigation of disease, is the palliation or cure, it is of the highest importance that the various remedies for Prolapsus should be considered in its treatment.

It would have been much better for suffering females had Prolapsus uteri never been entered in the catalogue of uterine diseases; for with the attention of the physician fixed exclusively on remedying the displacement; in the application of treatment, injury time and again has been done in those mechanical appliances intended to support the falling womb.

Suppose, as we have said, Prolapsus should result from chronic inflammation or other disease of the uterus, vagina or other neighboring parts, and to restore and keep in place the descended uterus, a pessary be placed in contact with the already irritated surfaces of these organs. Under such circumstances, does not every practical physician know that the radical disease from which the displacement has occured, will be aggravated? Pessaries, though useful in some cases of Prolapsus, are far from being admissible in a majority of instances, where this state of the organ is found. A support to the abdominal viscera, so as to prevent their weight upon the fundus of the uterus may be of more general use in uterine disease with a tendency to Prolapsus; for this may be instituted without detriment to the primary affection of the womb during its rational treatment.

